# Chinese Herbal Medicine Formula Shenling Baizhu San Ameliorates High-Fat Diet-Induced NAFLD in Rats by Modulating Hepatic MicroRNA Expression Profiles

**DOI:** 10.1155/2019/8479680

**Published:** 2019-12-14

**Authors:** Maoxing Pan, Yuanjun Deng, Chuiyang Zheng, Huan Nie, Kairui Tang, Yupei Zhang, Qinhe Yang

**Affiliations:** School of Traditional Chinese Medicine, Jinan University, Guangzhou 510632, China

## Abstract

**Objective:**

The purpose of present study was to investigate the potential mechanism underlying the protective effect of Shenling Baizhu San (SLBZS) on nonalcoholic fatty liver disease (NAFLD) by microRNA (miRNA) sequencing.

**Methods:**

Thirty male Wistar rats were randomly divided into a normal control (NC) group, a high-fat diet (HFD) group, and an SLBZS group. After 12 weeks, the biochemical parameters and liver histologies of the rats were assessed. The Illumina HiSeq 2500 sequencing platform was used to analyse the hepatic miRNA expression profiles. Representative differentially expressed miRNAs were further validated by qRT-PCR. The functions of the differentially expressed miRNAs were analysed by bioinformatics.

**Results:**

Our results identified 102 miRNAs that were differentially expressed in the HFD group compared with the NC group. Among those differentially expressed miRNAs, the expression levels of 28 miRNAs were reversed by SLBZS administration, suggesting the modulation effect of SLBZS on hepatic miRNA expression profiles. The qRT-PCR results confirmed that the expression levels of miR-155-5p, miR-146b-5p, miR-132-3p, and miR-34a-5p were consistent with those detected by sequencing. Bioinformatics analyses indicated that the target genes of the differentially expressed miRNAs reversed by SLBZS were mainly related to metabolic pathways.

**Conclusion:**

This study provides novel insights into the mechanism of SLBZS in protecting against NAFLD; this mechanism may be partly related to the modulation of hepatic miRNA expression and their target pathways.

## 1. Introduction

Nonalcoholic fatty liver disease (NAFLD) is defined as the pathological accumulation of fat in the liver without a history of excessive alcohol consumption. It encompasses a wide spectrum of conditions ranging from simple steatosis to nonalcoholic steatohepatitis (NASH) and may progress to advanced liver cirrhosis and hepatocellular carcinoma (HCC) [1, 2]. As a multifactorial disease with a complex pathogenesis, NAFLD is currently considered as the liver manifestation of the metabolic syndrome (MS). Excessive body weight, especially central obesity, insulin resistance, oxidative stress, mitochondrial dysfunction, glucose intolerance, dyslipidemia, and other metabolic syndromes are closely related to NAFLD [3–5]. With the ongoing epidemic of obesity, NAFLD has become a growing cause of chronic liver disease and the most common liver disease that influences approximately 25% of the population globally, and its prevalence is continuously increasing [6]. However, there are no specific pharmacological treatments for NAFLD thus far [7]. Lifestyle modification is still regarded as an important approach to slow the progression of NAFLD, but long-term adherence to lifestyle interventions is often difficult for patients to adhere to [8, 9]. Therefore, much effort has been focused on the development of novel prophylactic and therapeutic agents for NAFLD.

MicroRNAs (miRNAs) are a class of endogenous, noncoding, small-molecule, single-stranded RNAs that are widely involved in many basic biological activities, such as cell proliferation, differentiation, development, metabolism, and apoptosis [10]. There is some evidence that many miRNAs are key regulators of lipid metabolism and are involved in the pathogenesis of NAFLD [11]. For instance, previous studies have reported that miR-122 may regulate cholesterol and fatty acid metabolism [12, 13], and miR-21 has been demonstrated to play an important role in the hepatic inflammatory response caused by steatosis [14]. In addition, several other miRNAs have been found to be closely related to NAFLD, such as miR-33a/b, miR-34a, and miR-29 [15]. Therefore, it is necessary to further study the changes in the expression profile of microRNAs to provide a basis for exploring the particular mechanism of microRNAs in NAFLD.

Shenling Baizhu San (SLBZS) is a famous traditional Chinese medicine formula that has been used for hundreds of years for the treatment of digestive and metabolic disorders. More recently, accumulating evidence reveals that several bioactive ingredients of SLBZS, such as Ginsenoside Rb2, Ginsenoside Rg3, and glycyrrhizic acid, exert positive effects in protecting against NAFLD [16–18]. Our previous studies have demonstrated that SLBZS can protect against NAFLD by improving hepatic inflammation, inhibiting hepatic lipid accumulation, and thereby reducing hepatic steatosis [19, 20]. Based on the important role of miRNA in the pathogenesis of NAFLD, it is tempting to speculate that the protective effects of SLBZS against NAFLD may be related to the modulation of hepatic miRNA expression profiles. Therefore, in this study, we performed miRNA sequencing to systematically analyse the hepatic miRNA expression profiles in NAFLD rats induced by high-fat feeding, which aims to further understand the mechanism of protective effects of SLBZS on NAFLD.

## 2. Materials and Methods

### 2.1. Animals and Drugs

Thirty male Wistar rats weighing 160–180 g were obtained from Jinan Peng Yue Laboratory Animal Breeding Co., Ltd. (Jinan, Shandong, China). Animals were housed in separate cages in a specific pathogen-free (SPF) animal laboratory under controlled temperature (22 ± 2°C) and humidity (55 ± 5%) conditions with a 12 h light-dark cycle. Rats were allowed free access to water and standard chow for 1 week prior to the experiments. The experimental protocol was reviewed and approved by the Laboratory Animal Ethics Committee of Jinan University (Approval number: IACUC-20180412-04). All traditional Chinese medicines were in the form of formula granules and were purchased from Tian Jiang Pharmaceutical Co., Ltd., Jiangyin, China, and the composition of SLBZS includes 10 traditional Chinese medicines listed in Table 1.

### 2.2. Experimental Procedures

After one week of acclimation, the rats were randomly divided into 3 groups with 10 rats per group as follows: the normal control (NC) group, the high-fat diet (HFD) group, and the SLBZS group. The NC group was fed a normal diet (13% kcal from fat, 22% kcal from protein, 65% kcal from carbohydrate and no cholesterol), while the HFD group and SLBZS group were fed an HFD (37% kcal from fat, 22% kcal from protein, and 41% kcal from carbohydrate and 12 g/kg cholesterol) for 12 weeks. The SLBZS group was intragastrically administered SLBZS 30 g/kg once daily from week 7, while the NC group and HFD group were intragastrically administered an equal volume of distilled water. The diets were obtained from Trophic Animal Feed High-Tech Co., Ltd. Nantong, China. The drug dosage used in this study was based on that used in our previous studies [19, 20]. At the end of week 12, the body weight and nasoanal length were measured, and Lee's index was then calculated as body weight (g)^1/3^ × 1000/nasoanal length (cm). All rats were sacrificed using anaesthesia after 12 h of fasting. Blood samples were collected from the abdominal aorta immediately and then centrifuged at 1500 g for 10 min at 4°C. The supernatants were collected and stored at −80°C for biochemical analysis. The livers were rapidly removed and weighed to calculate the liver index (liver weight (g)/body weight (g) × 100%), and one part of each liver was fixed in 10% formalin for histological analysis. The remaining liver tissues were rapidly frozen in liquid nitrogen prior to storage at −80°C for subsequent analyses.

### 2.3. Biochemical Analysis in Serum and Liver Tissue

The serum levels of total cholesterol (TC), triglyceride (TG), high-density lipoprotein cholesterol (HDL-C), low-density lipoprotein cholesterol (LDL-C), alanine transaminase (ALT), and aspartate transaminase (AST) were determined by an automatic biochemical analyser (Rayto Life and Analytical Sciences Co., Ltd., Shenzhen, China). Serum free fatty acid (FFA) levels and hepatic TC and TG levels were measured using corresponding commercial kits (Jiancheng Bioengineering Institute, Nanjing, China).

### 2.4. Histological Analysis

Liver samples fixed with 10% formalin were dehydrated and embedded in paraffin. The paraffin-embedded liver samples were sectioned at a thickness of 5 *μ*m and then stained with haematoxylin and eosin (H&E). Frozen liver tissues embedded in optimum cutting temperature compound were sliced at a thickness of 8 *μ*m and then stained using an Oil Red O Stain Kit (Jiancheng Bioengineering Institute, Nanjing, China). The liver sections were observed under a light microscope.

### 2.5. Construction of Small RNA Libraries and Solexa Sequencing

Liver samples from three rats in each group were selected randomly and delivered to RiboBio Co., Ltd. (Guangzhou, China) for small RNA library construction and Solexa sequencing. The library construction process mainly includes the following steps: extracting the total RNA of the sample or the purified sRNA fragment, connecting the 3′ end and the 5′ linker, reverse transcription into cDNA, and then performing PCR amplification. Subsequently, the target fragment library was recovered by gelatinization, and the qualified library was sequenced on the machine. Solexa sequencing was performed using an Illumina HiSeq™ 2500 sequencing platform (Illumina, Inc., San Diego, CA, USA). Raw reads were filtered to obtain the clean reads. Differentially expressed miRNAs were determined by the EdgeR algorithm according to the criteria of |log 2(fold change)| ≥ 1 and *P* < 0.05.

### 2.6. Quantitative Reverse Transcription-Polymerase Chain Reaction (qRT-PCR) of miRNA

Total RNA was extracted from each sample by using a total RNA extraction reagent (Takara, Kusatsu, Japan), and was then reverse transcribed into cDNA with Mir-X miRNA First-Strand Synthesis kit (Takara, Kusatsu, Japan). The sequences of the miRNA-specific primers for real-time qPCR are listed in Table 2, and the reverse primer was the mRQ 3′ primer provided in Mir-X miRNA First-Strand Synthesis Kit. qRT-PCR was performed using SYBR Premix Ex Taq (Takara, Kusatsu, Japan) for selected miRNAs. The reaction volume was 25 *μ*L, and the qRT-PCR conditions were as follows: 30 s at 95°C, 40 cycles of 5 s at 95°C, and 30 s at 60°C, followed by a melting curve analysis step. Every sample was repeated three times, and relative quantification was performed by the 2^−ΔΔCq^ method. U6 was used as the endogenous control to normalize the data.

### 2.7. Bioinformatics Analysis

The three databases, namely, TargetScan, miRDB, and miRWalk, were used to predict the target genes of differentially expressed miRNAs. Only the overlapping genes in three databases were identified as the targets of differentially expressed miRNAs. Gene ontology (GO) was used to categorize the functions of differentially expressed miRNAs. Kyoto Encyclopedia of Genes and Genomes (KEGG) database analysis was used to identify the main biochemical pathways and signalling pathways in which differentially expressed genes participate.

### 2.8. Statistical Analysis

All data are presented as the mean ± standard deviation. The statistical analyses were performed using SPSS 20.0 for Windows. Significance was assessed by one-way ANOVA followed by Tukey's test or Games–Howell test for multiple comparisons. A *P* value less than 0.05 was considered statistically significant.

## 3. Results

### 3.1. SLBZS Reduced the Body Weight, Liver Weight, and Liver Index of HFD-Fed Rats

To investigate the effects of SLBZS on body characteristics, male Wistar rats were fed an HFD for 12 weeks

**Figure 1:**
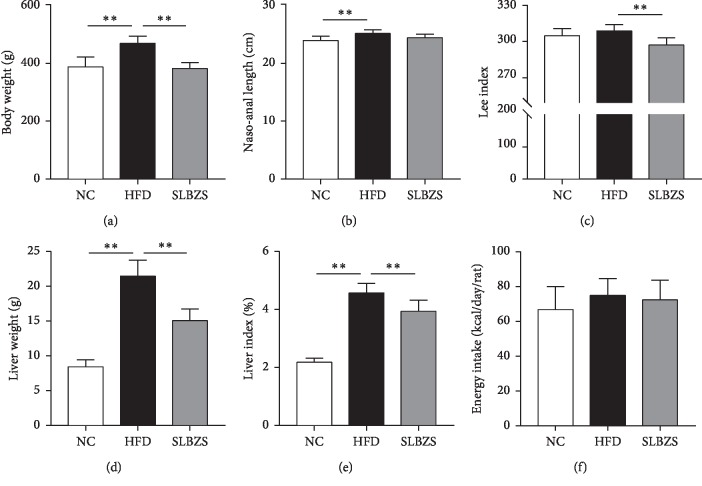
Effects of SLBZS on (a) body weight, (b) nasoanal length, (c) Lee index, (d) liver weight, (e) liver index, and (f) energy intake in HFD-fed rats. The values are presented as the mean ± standard deviations. Differences were assessed by ANOVA. ^*∗∗*^*P* < 0.01 vs the HFD group.

As shown in Figure 1, at the end of the 12 week experimental period, a high-fat diet significantly increased the body weight, nasoanal length, liver weight, and liver index of rats in the HFD group compared with those in the NC group (*P* < 0.01). With SLBZS administration, the body weight, Lee index, liver weight, and liver index of the rats in the SLBZS group were significantly lower than those of the rats in the HFD group (*P* < 0.01). However, no significant difference in energy intake was observed among the three groups.

### 3.2. SLBZS Improved the Biochemical Parameters of Blood in HFD-Fed Rats

**Figure 2:**
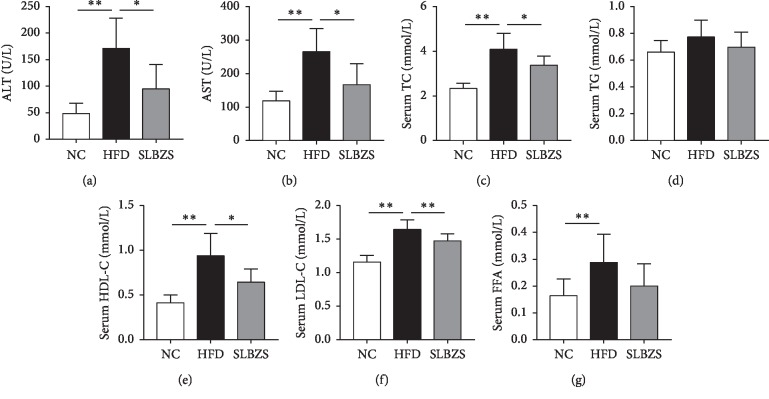
SLBZS improved the biochemical parameters: (a) ALT levels; (b) AST levels; (c) TC levels; (d) TG levels (e) HDL-C levels; (f) LDL-C levels; (g) FFA levels. Values are presented as the mean ± standard deviations. Differences were assessed by ANOVA. ^*∗*^*P* < 0.05 and ^*∗∗*^*P* < 0.01 vs the HFD group.

As shown in Figure 2, HFD-fed rats exhibited significantly higher serum levels of ALT, AST, TC, HDL-C, LDL-C, and FFA than the NC group (*P* < 0.01), and the SLBZS group had lower levels of ALT, AST, TC, HDL-C, and LDL-C than the HFD group (*P* < 0.05 or *P* < 0.01). The FFA levels exhibited a downward trend in the SLBZS group, although not in a significant manner. There were no significant differences among the groups in terms of serum TG levels. Overall, those results indicate that the abnormal biochemical parameters induced by an HFD were attenuated by SLBZS.

### 3.3. SLBZS Improved Hepatic Steatosis in HFD-Fed Rats

**Figure 3:**
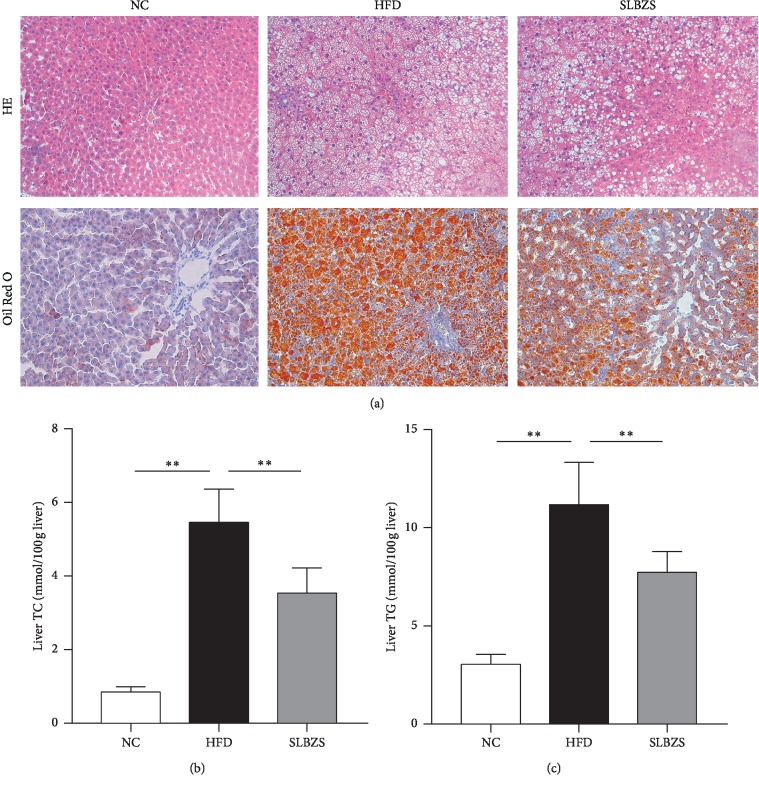
SLBZS attenuated hepatic steatosis: (a) representative H&E and oil red O images (magnification, 200×); (b) liver TC levels; (c) liver TG levels. Values are presented as the mean ± standard deviations. Differences were assessed by ANOVA. ^*∗∗*^*P* < 0.01 vs the HFD group.

For histological analyses, liver tissues were stained with H&E and Oil red O. As shown in Figure 3(a), the livers from rats on an HFD for 12 weeks showed typical fat deposition and hepatic steatosis, which was confirmed by excessive lipid droplets inside the cytoplasm with obvious swelling or even ballooning, implying that HFD feeding can induce NAFLD. There were no histological changes in the NC group, and the fat deposition and hepatic steatosis in the HFD group were significantly improved by SLBZS administration. As shown in Figures 3(b) and 3(c), consistent with the histological results, the rats in the HFD group had higher liver TC and TG levels than those in the NC group (*P* < 0.01), while the SLBZS group demonstrated significant reductions in their liver TC and TG levels compared with those in the HFD group (*P* < 0.01). These results indicate that SLBZS improved hepatic steatosis and lipid accumulation in HFD-fed rats.

### 3.4. SLBZS Systematically Modulated Hepatic miRNA Expression Profiles in HFD-Fed Rats

**Figure 4:**
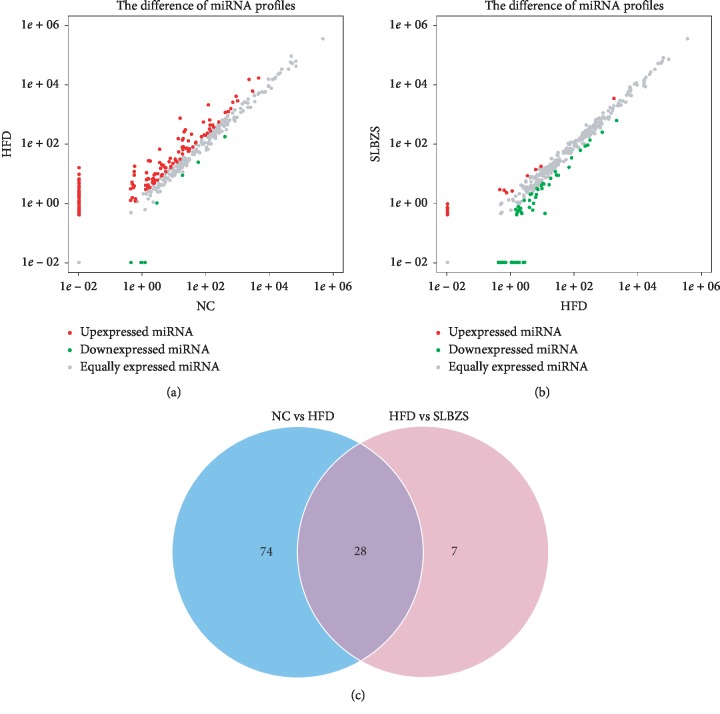
Cluster analysis of miRNAs expressed in HFD vs NC (a) and SLBZS vs HFD (b) and Venn diagram (c) of the differentially expressed miRNAs. In differentially expressed miRNA scatter plots, red indicates upregulated miRNAs, green indicates downregulated miRNAs, and grey indicates no significant changes in miRNA expression. The threshold set for significantly differential genes was |log 2(fold change)| ≥ 1 and *P* < 0.05.

**Figure 5:**
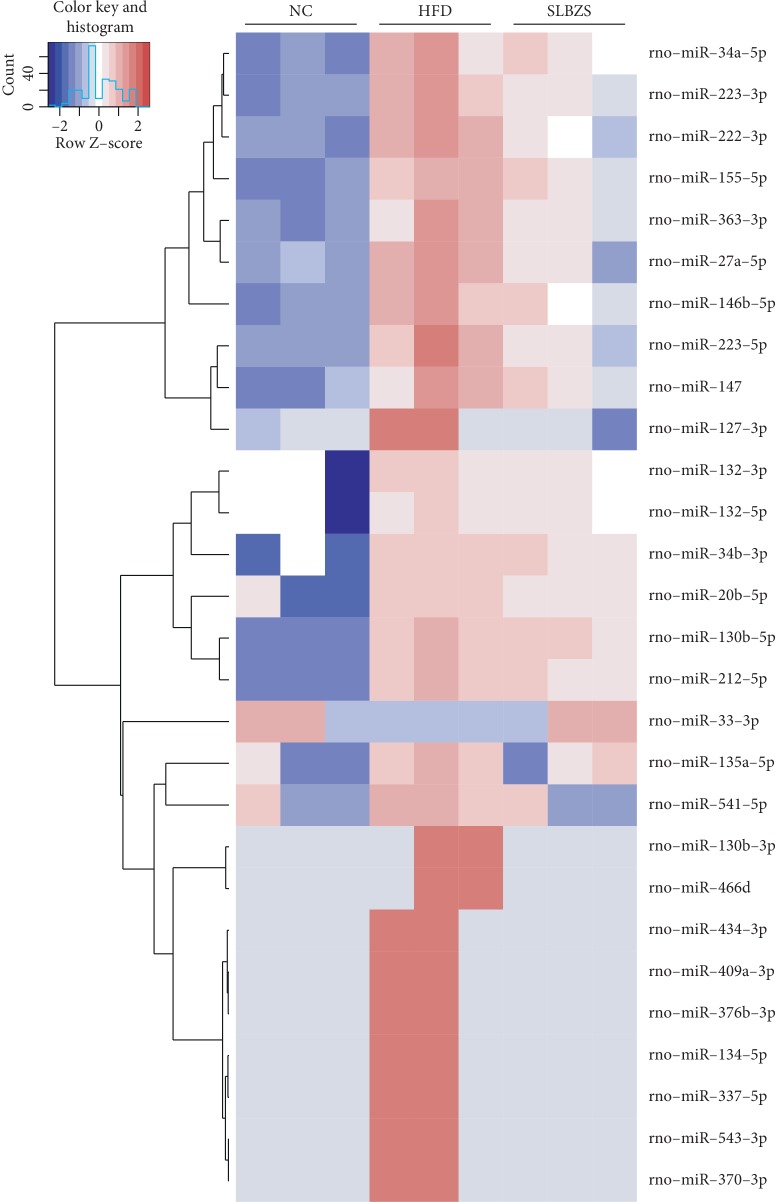
Heatmap and hierarchical clustering of 28 differentially expressed miRNAs in HFD-fed rats with SLBZS administration. Each row represents a miRNA, and each column represents a sample. The colour scale shown at the top illustrates the relative expression level of miRNAs; red represents a high relative expression level, and blue represents a low relative expression level.

We analysed the hepatic miRNA expression profiles in different groups. As shown in Figure 4, there were 102 differentially expressed miRNAs identified in the HFD group compared with the NC group (|log 2(fold change)| ≥ 1 and *P* < 0.05), 6 miRNAs were downregulated, and 96 differentially expressed miRNAs were upregulated. On the other hand, there were 35 differentially expressed miRNAs in the SLBZS group compared with those in the HFD group (|log 2(fold change)| ≥ 1 and *P* < 0.05); 30 miRNAs were downregulated and 5 differentially expressed miRNAs were upregulated. Interestingly, we found that the abnormal expression of 28 miRNAs in the HFD group was reversed by SLBZS administration (Figure 4). The 28 differentially expressed miRNAs are listed in Table 3, and cluster analysis of miRNA expression is shown in Figure 5. These results indicate that SLBZS systematically modulated hepatic miRNA expression profiles in HFD-fed rats, and these 28 miRNAs may be important factors in the reversion progression of NAFLD by SLBZS.

### 3.5. qRT-PCR Validation

**Figure 6:**
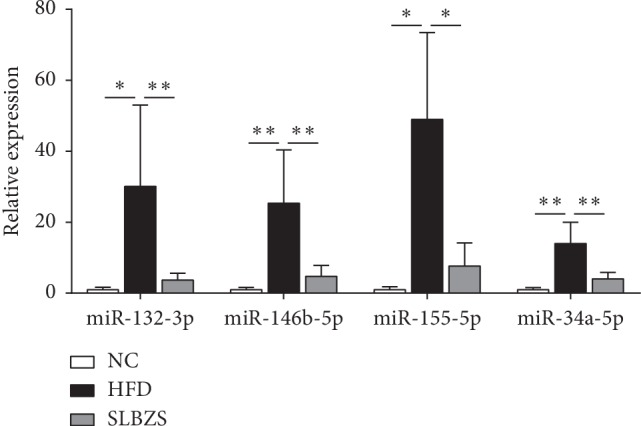
Relative expression of four selected miRNAs was quantified by qRT-PCR and normalized by U6 expression. Significance was assessed by one-way ANOVA followed by Tukey's multiple comparison tests. Data are presented as the mean ± SDs. ^*∗*^*P* < 0.05 and ^*∗∗*^*P* < 0.01 vs the HFD group.

To validate our findings based on miRNA sequencing, qRT-PCR was performed to assess the expression levels of 4 miRNAs overexpressed in the HFD group and reversed by SLBZS. As shown in Figure 6, miR-132-3p, miR-146b-5p, miR-155-5p, and miR-34a-5p were obviously upregulated after rats were fed a HFD for 12 weeks (*P* < 0.05 or *P* < 0.01). With SLBZS administration, the expression levels of the 4 miRNAs were significantly downregulated compared to those in the HFD group (*P* < 0.05 or *P* < 0.01). The qRT-PCR results were consistent with the miRNA sequencing data, thus indicating that the results of Solexa sequencing are reliable.

### 3.6. Prediction of miRNA Target Genes and Pathways Regulated by SLBZS

**Figure 7:**
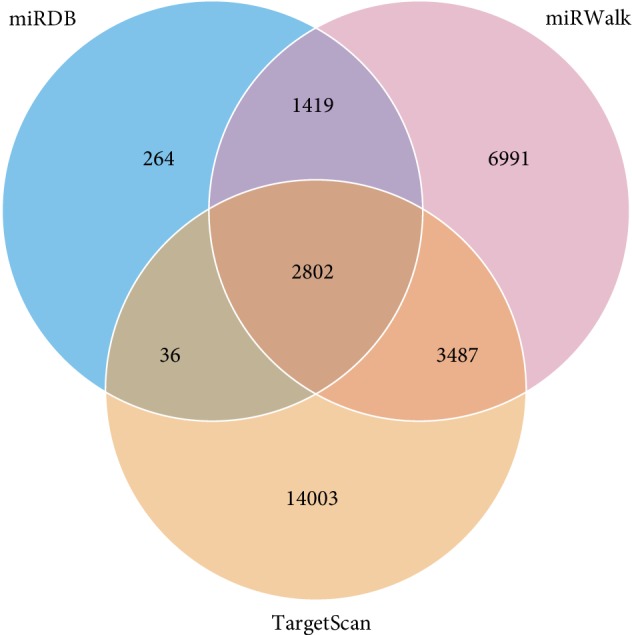
Venn diagram of the target gene counts from three different databases, taking the intersection of them finally resulted in 2802 target genes for further study.

**Figure 8:**
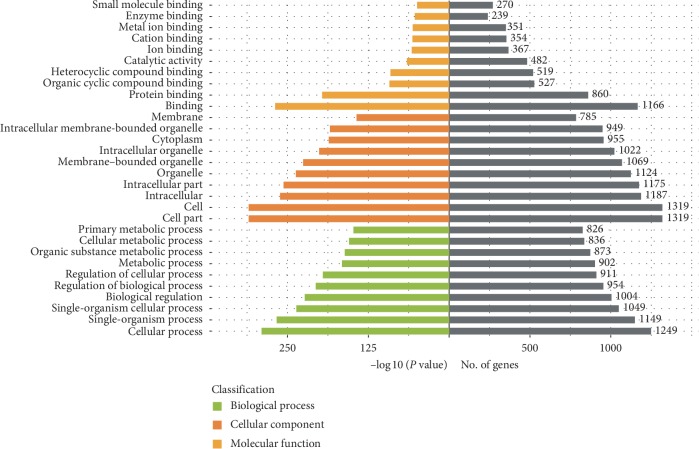
GO analysis for predicted targets of differentially expressed miRNAs (top 10 of fold enrichment).

**Figure 9:**
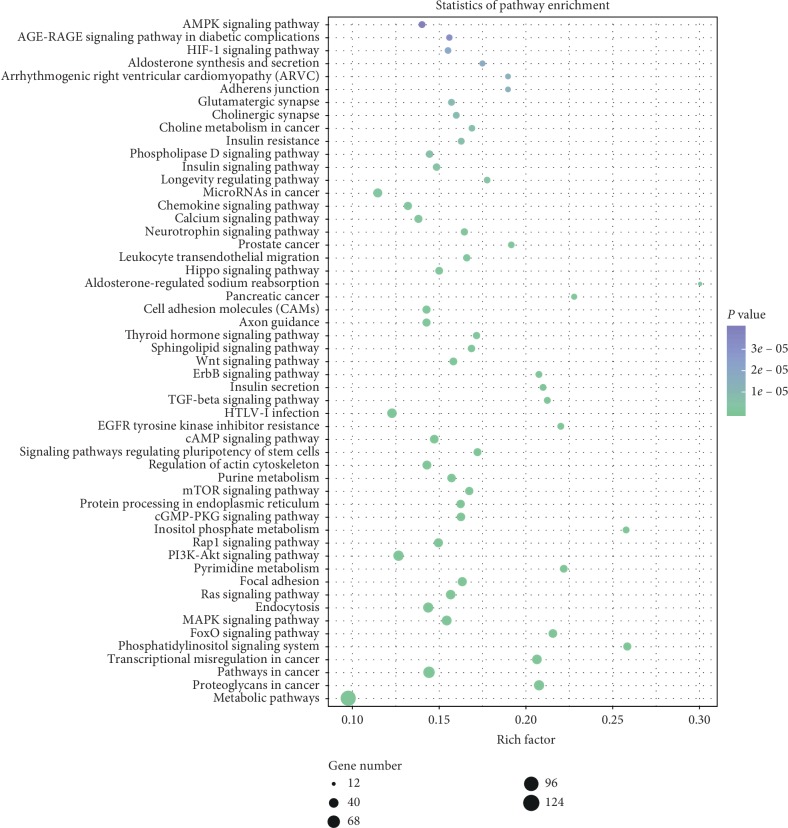
Pathway enrichment analysis of the target genes of differentially expressed miRNAs. The *x*-axis indicates the proportion of the enriched differential gene in the background gene of the pathway; the *y*-axis indicates the pathway name; point sizes indicate the number of differentially enriched genes; dot colours indicate the size of the *P* value (genes number ≥18 and *P* value <0.01).

**Figure 10:**
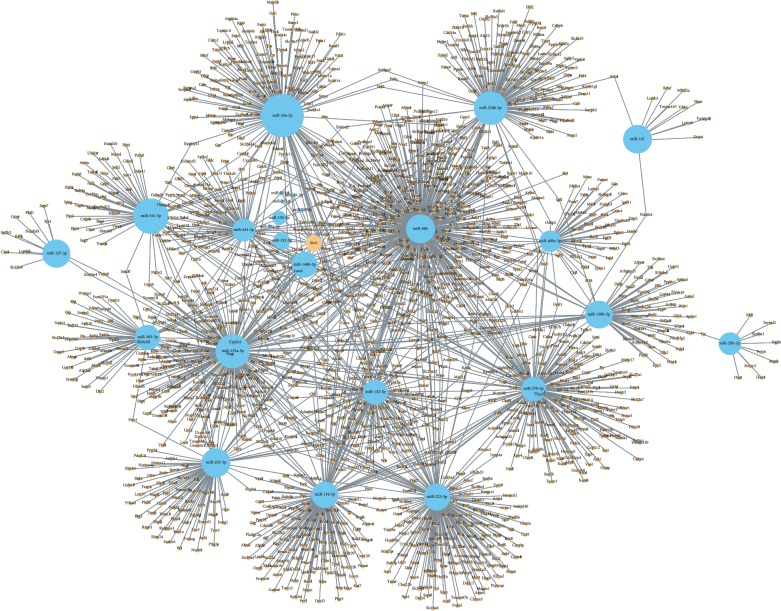
A network of the predicted target genes of differentially expressed miRNAs.

Three databases were used to predict the target genes of 28 differentially expressed miRNAs. Taking the intersection of predicted genes, 18 miRNAs were included and the other 10 miRNAs were excluded. Finally, 2802 target genes were predicted to be the targets of 18 differentially expressed miRNAs from three different databases (Figure 7). To predict the function of the differentially expressed miRNAs, the GO function annotation was used to provide annotations for the candidate target genes in terms of biological processes, cellular components, and molecular functions. We found that “cellular process,” “cell part,” “cell,” and “binding” were the most significantly enriched in each category (Figure 8). We used KEGG pathway annotation to identify the signal transduction and disease pathways controlled by the involved miRNAs, and we found that the most enriched pathways were as follows: metabolic pathways (containing 125 genes), PI3K-Akt signalling pathway (containing 44 genes), MAPK signalling pathway (containing 39 genes), FoxO signalling pathway (containing 29 genes), cAMP signalling pathway (containing 29 genes), mTOR signalling pathway (containing 26 genes), insulin signalling pathway (containing 21 genes), insulin resistance (containing 18 genes), and AMPK signalling pathway (containing 18 genes) (Figure 9). Moreover, the network of miRNA target gene interactions was visualized. It is apparent that miR-155-5p, miR-146b-5p, miR-132-3p, and miR-34a-5p share many targets, suggesting they have a common role in many pathways (Figure 10).

## 4. Discussion

NAFLD is one of the most prevalent chronic liver diseases in the world, and the aetiology of the disorder remains obscure. A suitable animal model that faithfully recapitulates the pathophysiology of NAFLD could provide valuable means to investigate its pathogenesis. In the present study, a 12-week HFD feeding successfully induced a rat NAFLD model, which manifested the pathogenesis and histopathological characteristics of human NAFLD as previously described [21]. Previous studies have reported that medicinal plants and bioactive natural compounds could also potentially improve NAFLD treatment with an acceptable safety [22]. This study was able to confirm the effect of SLBZS in the amelioration of HFD-induced NAFLD rats in the following ways. First, weight loss is the key to improvement in the histopathological features of NAFLD, so any treatment recommendations include weight loss as background therapy [23]. We demonstrated that SLBZS significantly reverted the body weight, liver weight, liver index, and Lee index gain in HFD-induced NAFLD rats. Second, lipid metabolism disorder and hepatocyte injury are associated with the development of fatty liver [24]. We confirmed that SLBZS can improve biochemical parameters in HFD-induced NAFLD rats. The administration of SLBZS plays a protective role in improving the abnormal serum lipid profiles and liver transaminases in HFD-fed rats. Therefore, SLBZS may improve metabolic disorders and protect liver function to achieve the anti-NLFLD effects. Third, hepatic lipid accumulation is one of the crucial pathophysiological mechanisms for NAFLD [25], and a liver biopsy is considered the “gold standard” for clinical diagnosis and stage, followed by histological analysis [26]. H&E and oil red O staining were used to measure the histological morphologies of the liver tissues. The histology examinations suggest that SLBZS may attenuate hepatic steatosis in HFD-fed rats. Furthermore, SLBZS can reduce hepatic lipid accumulation as evidenced by reductions in liver TC and TG. Collectively, these data demonstrated that SLBZS was effective in protection against NAFLD in rats.

The miRNA expression profile changes in NAFLD progression have been amply demonstrated in animal and human studies [27–29]. Although SLBZS is known to have potent anti-NLFLD effects, its underlying mechanism of action on miRNA has not been previously studied. To identify the miRNA expression profiles in an HFD-induced steatotic animal model with or without SLBZS administration, we used Solexa sequencing of miRNAs and qRT-PCR to validate the selected, abnormally expressed miRNAs. The data obtained by Solexa sequencing are more accurate and sensitive than the microarray platform [30]. In the present study, by analysing miRNA expression profiles, we found 109 miRNAs displayed differential expression patterns among the three groups, demonstrating a potent effect of SLBZS in globally altering hepatic miRNA expression. Moreover, we found that SLBZS reversed the abnormal expression of 28 miRNAs in the HFD group, among which miR-155-5p, miR-146b-5p, miR-132-3p, and miR-34a-5p were the most notable. With validation by qRT-PCR, we further confirmed that miR-155-5p, miR-146b-5p, miR-132-3p, and miR-34a-5p were increased in HFD-induced rats, and this increase was reversed by SLBZS administration. Therefore, miR-155-5p, miR-146b-5p, miR-132-3p, and miR-34a-5p and their target genes may be, at least partly, involved in the mechanism by which SLBZS protects against NAFLD.

Indeed, previous studies have shown that miR-155 plays a key role in the liver lipid metabolism and was found to be upregulated in NASH [31, 32]. Furthermore, a miR-155 deficiency has been shown to reduce steatosis and fibrosis [33]. A similar dysregulation in the expression of miR-132, miR-146b, and miR-34a has been reported in NAFLD. miR-132 was considered a key regulator of hepatic lipid homeostasis, and its overexpression showed a severe fatty liver phenotype in transgenic mice; hence, lowering its level may be a treatment for hepatic steatosis [34]. Several studies have shown that miR-146b and miR-34a are significantly elevated in animal models and NAFLD patients [35–37]. The present study showed the alterations of the miR-132, miR-146b, and miR-34a levels, which seem to be consistent with these findings. Notably, silent information regulator of transcription 1 (SIRT1), a key mediator of energy metabolism among multiple signalling pathways and a potential therapeutic target for the treatment of NAFLD [38, 39], appears to be a common direct target of miR-132, miR-146b, and miR-34a. Several studies also suggest that miR-132, miR-146b, and miR-34a could regulate SIRT1 expression [40, 41], and bioinformatics analysis supported this view as well (Figure 10). Therefore, the alteration of SIRT1 and its target pathways by regulating the miRNA expression profile may be one of the important mechanisms of SLBZS against NAFLD.

Furthermore, to further explore and depict the differentially expressed miRNAs functions, we predicted the target genes of the 28 miRNAs reversed by SLBZS and constructed miRNA target genes networks. A total of 2802 target genes were screened from the intersection of three databases, and GO and KEGG pathway analyses were performed to categorize 2802 target genes for 28 differentially expressed miRNAs. The results show that these genes are involved in diverse important signalling pathways, such as metabolic pathways, the PI3K-Akt signalling pathway, the MAPK signalling pathway, the FoxO signalling pathway, the cAMP signalling pathway, the mTOR signalling pathway, the insulin signalling pathway, and the AMPK signalling pathway. As expected, most of the target pathways had been reported to be closely associated with the progression of NAFLD [42–44]. For instance, previous studies have shown that HFD-induced liver steatosis was associated with depressed mRNA expression of AMPK-PGC-1*α* signalling components, nuclear respiratory factor-2 (NRF-2), and *β*-ATP synthase [45]. Supporting these findings, in our earlier study, we demonstrated that SLBZS might exert a significant anti-inflammatory effect in the Kupffer cells of NASH rats induced by an HFD through the suppression of the p38 MAPK pathway [19]. Of note, the FoxO signalling pathway, the mTOR signalling pathway, and the AMPK signalling pathway, the three notable pathways related to NAFLD, are likely to be involved in SIRT1-mediated autophagy. Several studies have demonstrated that autophagy induced by the AMPK/mTOR pathway can improve liver lipid accumulation [46], and SIRT1-FoxO signalling pathway-induced autophagy may serve as a critical role in protecting against NAFLD [47]. In addition, alleviating pro-lipogenic, pro-inflammatory, and the oxidative stress induced by HFD is an important way to resist NAFLD, including modulation the activity of the transcription factors PPAR-alpha, SREBP-1c, Nrf2, and NF-*κ*B [48–50]. Emerging evidence suggests that inflammation, oxidative stress and autophagy are closely associated with the development of NAFLD [51, 52]. Thus, the role of SLBZP in NAFLD deserves further exploration by modulating the miRNA profile to act on the autophagy pathway, thereby alleviating pro-lipogenic, proinflammatory and oxidative stress.

We have to note that there remain some limitations in the present study. First, the amount of Solexa sequencing samples was small, which might affect the accuracy and comprehensiveness of the miRNA expression profiles. Second, there is a lack of support for in vitro experiments, which may affect the reliability of the results. Third, the function of these differentially expressed miRNAs and their target genes in the development and pathogenesis of NAFLD require further biological experiments to clarify them.

## 5. Conclusion

To the best of our knowledge, this study is the first to demonstrate that HFD-induced steatosis in rats resulted in a global alteration in hepatic miRNA expression, which was significantly reversed by SLBZS administration. Among these differentially expressed miRNAs, miR-155-5p, miR-146b-5p, miR-132-3p, and miR-34a-5p were four important altered miRNAs found in the miRNA profiling study and confirmed by qRT-PCR. Bioinformatics analysis can provide further research ideas for the mechanism of the effects of SLBZS on NAFLD. Thus, the study provides novel insight into the mechanism of SLBZS in protecting against NAFLD, which may be partly related to modulation of hepatic miRNA expression and their target pathways.

## Figures and Tables

**Table 1:** Composition of SLBZS.

Chinese name	Plant	Part used	Proportion
Ren Shen	*Panax ginseng* C. A. Mey.	Root	5
Fu Ling	*Poria cocos* (Schw.) Wolf.	Sclerotium	5
Baizhu	*Atractylodes macrocephala* Koidz.	Rhizome	5
Shan Yao	*Dioscorea opposita* Thunb.	Rhizome	5
Bai Bian Dou	*Dolichos lablab* L.	Seed	4
Lian Zi	*Nelumbo nucifera* Gaertn.	Seed	3
Yi Yi Ren	*Coix lacryma-jobi* L.	Kernel	3
Zhi Gan Cao	*Glycyrrhiza uralensis* Fisch. Ex DC.	Root and rhizome	3
Jie Geng	*Platycodon grandiflorus* (Jacq.) A. DC.	Root	2
Sha Ren	*Amomum villosum* Lour.	Fruit	2

**Table 2:** miRNA primer sequences for qRT-PCR analysis.

miRNA	miRNA sequences	Mirbase	Primer sequence (5′–3′)
rno-miR-155-5p	UUAAUGCUAAUUGUGAUAGGGGU	MIMAT0030409	TAATGCTAATTGTGATAGGGGT
rno-miR-146b-5p	UGAGAACUGAAUUCCAUAGGCUGU	MIMAT0005595	GAACTGAATTCCATAGGCTGT
rno-miR-132-3p	UAACAGUCUACAGCCAUGGUCG	MIMAT0000838	CAGTCTACAGCCATGGTCG
rno-miR-34a-5p	UGGCAGUGUCUUAGCUGGUUGU	MIMAT0000815	GCAGTGTCTTAGCTGGTTGT

**Table 3:** SLBZS reversed 28 differentially expressed miRNAs in the HFD group relative to the NC group.

No.	miRNA	Expression type		log2 (fold change)		*P* value	
		HFD/NC	SLBZS/HFD	HFD/NC	SLBZS/HFD	HFD/NC	SLBZS/HFD
1	rno−miR−155−5p	Up	Down	5.6137	−1.5706	<0.001	<0.001
2	rno−miR−146b−5p	Up	Down	4.1624	−1.7567	<0.001	<0.001
3	rno-miR−34a−5p	Up	Down	3.7695	−1.1676	<0.001	<0.01
4	rno−miR−223−3p	Up	Down	3.6776	−1.4905	<0.001	<0.001
5	rno−miR−363−3p	Up	Down	3.5173	−1.3068	<0.001	<0.001
6	rno−miR−132−3p	Up	Down	4.1498	−1.6179	<0.001	<0.01
7	rno−miR−27a−5p	Up	Down	2.0672	−1.239	<0.001	<0.01
8	rno−miR−222−3p	Up	Down	2.6311	−1.3314	<0.001	<0.001
9	rno−miR−223−5p	Up	Down	4.2747	−2.0249	<0.001	<0.001
10	rno−miR−147	Up	Down	3.9297	−1.5527	<0.001	<0.01
11	rno−miR−127−3p	Up	Down	1.5407	−1.807	<0.05	<0.001
12	rno−miR−132−5p	Up	Down	3.0142	−1.2622	<0.01	<0.05
13	rno−miR−34b−3p	Up	Down	4.9801	−1.3596	<0.001	<0.05
14	rno−miR−20b−5p	Up	Down	4.0177	−1.163	<0.01	<0.05
15	rno−miR−130b−5p	Up	Down	10.6116	−1.9031	<0.001	<0.01
16	rno−miR−212−5p	Up	Down	9.8709	−1.5994	<0.001	<0.05
17	rno−miR−33−3p	Down	Up	−6.8908	6.5546	<0.01	<0.05
18	rno−miR−135a−5p	Up	Down	3.4632	−2.3622	<0.05	<0.05
19	rno−miR−541−5p	Up	Down	4.4179	−4.6897	<0.01	<0.001
20	rno−miR−130b−3p	Up	Down	7.2062	−7.2062	<0.01	<0.01
21	rno−miR−466d	Up	Down	6.705	−6.705	<0.05	<0.01
22	rno−miR−434−3p	Up	Down	8.0334	−8.0334	<0.01	<0.01
23	rno−miR−409a−3p	Up	Down	8.0571	−8.0571	<0.01	<0.01
24	rno−miR−376b−3p	Up	Down	7.9069	−7.9069	<0.01	<0.01
25	rno−miR−134−5p	Up	Down	7.4865	−7.4865	<0.01	<0.01
26	rno−miR−337−5p	Up	Down	7.3099	−7.3099	<0.01	<0.01
27	rno−miR−543−3p	Up	Down	6.9268	−6.9268	<0.05	<0.01
28	rno−miR−370−3p	Up	Down	6.8287	−6.8287	<0.05	<0.05

## Data Availability

The datasets used and analysed during the current study are available from the corresponding author upon reasonable request.
